# Age-Related Differences in Reading Negative Texts on COVID-19

**DOI:** 10.1192/j.eurpsy.2023.1249

**Published:** 2023-07-19

**Authors:** A. Popov, S. Tukaiev, V. Rizun, Y. Havrylets, D. Ivaskevych, A. Petrenko-Lysak, Y. Yachnik

**Affiliations:** 1 Department of Electronic Engineering, Igor Sikorsky Kyiv Polytechnic Institute, Kyiv, Ukraine; 2 Faculty of Communication, Culture, and Society, Institute of Public Health, Università della Svizzera italiana, Lugano, Switzerland; 3 Institute of Journalism, Taras Shevchenko National University of Kyiv, Kyiv, Ukraine; 4 Department of Branch Sociology, Faculty of Sociology, Taras Shevchenko National University of Kyiv, Kyiv, Switzerland; 5 University clinic, Taras Shevchenko National University of Kyiv, Kyiv, Ukraine

## Abstract

**Introduction:**

The worldwide COVID-19 pandemic leads to the development of stress disorders, increased anxiety in the society. One of the strongest factors leading to the development of anxiety, stress in society during a pandemic is the Mass Media. The mechanisms of stressogenic effects of Mass Media remain not completely clear.

**Objectives:**

The aim of this study was to evaluate age-specific characteristics of gaze behaviour related to the perception of anxiety-provoking information.

**Methods:**

189 volunteers took part in the study (164 participants aged between 17 and 22 years old (students, control group), 25 people aged between 59 and 71 (experimental group)). Participants were asked to fill in Psychological Stress Measure (PSM-25), Patient Health Questionnaire (PHQ-9), and Generalised Anxiety Disorder Assessment (GAD-7) questionnaires in order to determine their levels of stress, depression, and anxiety. The second stage of the research was an eye-tracking study of text perception - we analysed eye-tracking data during the text perception by using web-eye tracking (EyePass).

**Results:**

To identify the relevance of COVID-19 information, we compared the perception of positive, neutral and negative texts and detected rather a negativity bias than attraction to the positive or neutral stimuli: fixations positive vs negative (p-value < 0.01), positive vs neutral (p-value < 0.01), neutral vs negative (p-value < 0.01). Under the competitive conditions (higher relevance of negative information during Pandemic) the perception of negative text is characterized by attentional priority. Moreover, it should be taken into account that highly anxious participants showed a negativity bias than attraction to the positive stimuli, and this is typical during quarantine.

**Conclusions:**

Therefore, participants showed a negativity bias than attraction to the positive stimuli, and this is typical during quarantine. There are significant age-related differences in gaze behavior while reading text with negative text elements. While the origin of these differences between older and younger adults remains unknown, further research may provide more evidence about the origin of this effect.

**Disclosure of Interest:**

None Declared

